# A Review of Welfare Indicators of Indoor-Housed Dairy Cow as a Basis for Integrated Automatic Welfare Assessment Systems

**DOI:** 10.3390/ani10081430

**Published:** 2020-08-15

**Authors:** Lisette M.C. Leliveld, Giorgio Provolo

**Affiliations:** Department of Agricultural and Environmental Sciences, Università degli Studi di Milano, 20133 Milano, Italy; Giorgio.Provolo@unimi.it

**Keywords:** precision livestock farming, data integration, animal welfare, dairy cattle, health monitoring, *Bos taurus*

## Abstract

**Simple Summary:**

Many techniques have been developed to measure single indicators of reduced welfare in farm animals, such as changes in the walking pattern to detect lameness in dairy cows. However, there is still a need to combine these single measurements to get a more complete picture of the wellbeing of an animal. Based on a literature review on dairy cow welfare, this review provides a basis for the development of an integrated automatic system to assess the welfare of dairy cows on the farm. It provides an overview of the main welfare issues for dairy cows, such as lameness, heat stress, or pain and of the most established indicators that could help to detect these welfare issues on the farm. We found that there are several indicators, such as reduced feed intake, that are common to most welfare issues and that are therefore suitable to detect reduced welfare in general, while other indicators mainly identify one welfare issue, such as increased respiratory rate, as an indicator of heat stress. Combining these different types of indicators would provide a good basis to develop an integrated automatic system that could assist farmers in the detection of reduced welfare on their farms.

**Abstract:**

For on-farm welfare assessment many automatic methods have been developed to detect indicators of reduced welfare. However, there is still a need to integrate data from single sources to obtain a complete picture of the welfare of an animal. This review offers a basis for developing integrated automatic systems to assess dairy cow welfare by providing an overview of the main issues that challenge cow welfare (e.g., lameness) and of well-established indicators that could detect these issues on the farm. Based on a literature review of 4 reviews on cow welfare in general and 48 reviews on single welfare issues, we identified 18 different major welfare issues and 76 matching indicators that could be detected automatically on the farm. Several indicators, e.g., feed intake, showed a consistent association with welfare across many different issues. Although some of these indicators are discussed critically, this means there are many indicators that potentially could detect reduced welfare in general. Other types of indicators could detect one specific welfare issue, e.g., increased respiratory rate for heat stress. These different types of indicators combined provide a basis to develop integrated automatic systems that ultimately would help farmers to detect welfare problems at an early stage.

## 1. Introduction

The need to improve the welfare of dairy cows has for a long time been underrated, both by the public [1] and by the legislators [2]. For instance, in contrast to other livestock groups—such as veal calves, pigs, and poultry—there is no specific EU directive for dairy cows. However, a recent report by the EU Parliament’s Directorate-General for Internal Policies stated that dairy cow welfare may be considered to be the second greatest animal welfare problem in the EU [2,3]. Therefore, there is an urgent need to improve cow welfare on the farm, for which an accurate assessment is the first step. To assess the welfare of farm animals often three important questions are raised: (1) Is the animal functioning well? (2) Is the animal feeling well? (3) is the animal able to live a reasonably natural life? (e.g., [4,5,6]). While opinions differ on which of these (partially overlapping) concerns is most important, it is commonly accepted that none of these three concerns can fully account for all aspects related to animal welfare [4]. For instance, tied-up dairy cows may have considerably lowered risks of hoof problems and lameness, but, due to lack of movement and social contact, would still be considered to have bad welfare [5]. Inversely, certain health risks (e.g., ammoniac exposure) may not be recognized by the animal [4,7]. This highlights the importance of assessing animal welfare as a whole, by taking into account different welfare aspects and integrating these into a complete picture. In farm practice, this could be achieved by integrating data from different welfare indicators to provide an overall evaluation of the animal unit [8]. In small-scale farms, the farmer would naturally perform such data integration by observing different welfare indicators during daily routine or close examination of each animal and by combining these different indicators to make an assessment. However, sub-clinical health issues are often not detected by farmers [9,10]. Moreover, due to the increase in farm size and the accompanying automation of farming, individual examinations by the farmer have become less frequent [10,11], which further impairs the on-farm detection of reduced welfare. In order to ensure good welfare on big modern farms, many strategies in the field of precision livestock farming (PLF) have been developed to measure welfare indicators automatically (for reviews see e.g., [9,12,13]). In general, automatic detection methods focus on one of two types of indicators, i.e., environment-based indicators and animal-based indicators [14]. For instance, in the assessment of heat stress both environment-based indicators, such as ambient temperature, and animal-based indicators, such as respiratory rate, could be measured (e.g., [15,16]). While studies often focus on testing the performance of one automatic detection method, several studies have found that integrating data from different detection methods improves the detection performance (e.g., [17,18]; reviewed by [19]) and commercial systems that integrate several parameters, such as RumiWatch (Itin + Hoch GmbH, Liestal, Switzerland [20]) and Cow Manager SensOor (Agis Automatisering BV, Harmelen, the Netherlands [21]), have been suggested to outperform systems with less or one parameter [22,23]. However, there is still a need to integrate data from many systems to into one system that would be able to make an overall assessment of the welfare of an animal (e.g., [24,25]).

Integrated welfare assessment systems would allow to capture the biological complexity of some disorders [26] and allow to draw conclusions that are not apparent from single sources [25]. We like to extend on this argumentation by suggesting that, compared to single sources, there are two main opportunities for an integrated system: (1) to allow a broader assessment of reduced welfare of an animal and (2) to differentiate between different welfare issues (e.g., between lameness and heat stress). To meet these opportunities, however, a careful selection of suitable indicators is needed. The first step in this selection is to obtain an overview of how the three different welfare concerns (biological function, affective state, and natural life) are challenged in the concerned livestock group (i.e., which are the main welfare issues?). The second step is then to carefully select reliable indicators of these identified welfare issues (i.e., which are the indicators?). In the selection of the indicators two requirements should be met (matching the two opportunities mentioned above). These are (1) to accurately detect as many incidences of reduced welfare as possible, while keeping the cost low and (2) to differentiate between the different welfare issues to give a clear advice to the farmer. Requirement 1 ensures that the farmer is informed that something is wrong, but not about what is wrong. Requirement 2 ensures that the farmer is informed about what is wrong, if it concerns a pre-defined welfare issue. However, it may not provide any guidance in case of new or relatively uncommon welfare issues.

The aim of this review is to provide a basis for developing integrated automatic systems to assess dairy cow welfare by, first, obtaining an overview of cow welfare and, second, selecting the welfare indicators that need to be measured. Further steps in the development of integrated automatic assessment systems, such as an evaluation of the available automatic detection techniques, are not the focus of the current review, since these would require a separate review. To achieve this, this review will first provide a short overview of the main issues that challenge the welfare of dairy cows (e.g., lameness). Second, it will aim to identify well-established indicators of these welfare issues that are suitable for an integrated automatic assessment system. The focus, thereby, will be on indicators that could be measured indoors, since it would be easier to apply integrated automatic assessment systems in this fixed environment. To understand which of these indicators may be most suitable for an integrated automatic assessment system we evaluate them according to the two requirements described in the previous section. For requirement 1, we will focus on indicators that have an association with dairy cow welfare across the different welfare issues (i.e., associations with many different welfare issues) in a clear direction (i.e., agreement across the references for either a positive or negative association; here referred to as *generalist indicators*). For requirement 2, we will focus on two possible strategies. One is to combine several generalist indicators to differentiate between single welfare issues. Another strategy is to select indicators that have an association with single welfare issues (e.g., lameness) that are well-established (i.e., supported by many references) in a clear direction (i.e., agreement across the references for either a positive or negative association) and that have no similar association with other single welfare issues (here referred to as *specialist indicators*). The review will close with a discussion of further considerations for optimizing an integrated automatic assessment system.

## 2. Literature Research

The main welfare concerns that dairy cows are dealing with were extracted from two welfare reports, the Welfare Quality Assessment Protocol [27], which is compiled by a network of applied ethologists and a report from the European Food Safety Authority [28]. In addition, welfare issues were also extracted from two books focusing on cow welfare [5,29]. 

Next, reviews on each single welfare issue, as well as on the three main welfare concerns (biological functioning, affective state, and natural lives), were searched using two search engines (Scopus and Web of Science). The search terms were cattle/cow + each welfare issue (including alternative terms; see Table S1 for full list of search terms) in the title. The titles and abstracts of the resulting list of reviews (476 in Web of Science and 669 in Scopus) were then scanned to determine suitability for this review. A review was only selected if it mentioned in the title or abstract that it aimed to provide an overview of indicators or if it mentioned more than one indicator that could potentially be assessed automatically on the farm (meaning not requiring a physical examination). Exclusion criteria were: abstract could not be found (if the title did not provide enough information), not written in a language that could not be understood by the authors (i.e., other than English, German, Dutch, or Italian), a focus only on specific aspects of a welfare issue (e.g., reoccurrence of mastitis; however, sub-issues that were mentioned in the four sources, e.g., specifically mentioned infectious diseases, were included), a specific focus on other cattle groups than dairy cows (e.g., beef cattle or calves) or on pasture-held cattle (since the focus of this paper is on indoor housed cattle). This selection resulted in a total of 116 review finds; of these, 34 were overlapping between the two search engines and six review finds could not be obtained, resulting in a total of 76 available review finds. Finally, five reviews overlapped between welfare issues, meaning that the total number of obtained reviews was 71.

For each welfare issue the two books on cow welfare [5,29] and reviews were read in a reverse chronological order until either the limit of a maximum of 5 reviews or the limit of more than 200 collected references was reached. There were exceptions to the limit of five reviews, namely if a review provided zero referenced indicators on its respective welfare issue, the limit was extended by 1 review (if available). In total 48 reviews were read (4 of these reviews covered two welfare issues; see Table 1). Only indicators that could potentially be measured automatically in the barn environment (either with already existing techniques or by adapting these techniques), that were not a consequence of conscious decision making by the famer (e.g., diet composition and stall design) and that were supported by references were selected (in case of multiple references, each reference was counted separately). Since the overview serves to provide the basis for an integrated automatic assessment system to provide early warnings to the farmer, any indicators related to mortality and reproductive performance were excluded, since these indicators are only detectable on the long term. It was noted whether the direction of association was positive (i.e., increasing with an increasing occurrence or severity of the welfare issue) or negative (i.e., decreasing with an increasing occurrence or severity of the welfare issue). Because nearly all welfare issues reflect reduced welfare (positive emotions excepted), this means that indicators with positive associations with these welfare issues also reflect reduced welfare, while indicators with negative associations reflect improved welfare. The supporting references were noted to avoid double counting overlapping references between reviews. A reference could support associations of an indicator with more than one welfare issue (e.g., an arched back as an indicator of lameness and pain), but only if these associations are specifically mentioned in the review. Also, a reference could be included more than one time as a support for an association between an indicator and a welfare issue, if the reference provided support for different sub-types within a welfare issue (e.g., for different types of infectious diseases). However, different measures of the same type of indicator were combined to reduce complexity and to avoid an overrepresentation of single studies (e.g., lying time and lying frequency were combined in the indicator ‘lying behavior’).

To identify indicators that allow the detection of reduced welfare in general (generalists), the indicators were ranked according to the number of welfare issues they had been reported to have associations with. In addition, to determine whether an indicator has a clear pattern of association with cow welfare across the different issues, the number of references indicating a positive or negative association were compared. A binomial test was performed, using the FREQ procedure of SAS (version 9.4; SAS Institute Inc., Cary, NC, USA), to determine if there is consensus on the direction of association (by comparing the number of references indicating a positive or negative association), i.e., if the majority of references suggest the same direction. To identify indicators that allow to set single welfare issues apart from the other issues (specialists), the number of references indicating a positive or negative direction were compared for each association between an indicator and a single welfare issue, using a binomial test. Associations were compared between welfare issues to identify unique associations, which means that the indicator had no similar association with another welfare issue.

## 3. Cow Welfare

Using the four sources described in Section 2 [5,27,28,29], we identified eight welfare issues that primarily relate to biological functioning. It is however acknowledged that these welfare issues may also relate to the concerns of affective state and natural living. These issues were: undernutrition (or negative energy balance), dehydration, heat stress, injuries, lameness, infectious diseases (including mastitis, metritis, and tuberculosis), metabolic disorders (including ketosis and milk fever), reproductive disorders (including dystocia). Mortality was also mentioned [5,28]. However, since automatic assessment systems are aimed at preventing mortality, rather than detecting it, this welfare issue is not further addressed in this review. Lameness is often described as one of the major health disorders in dairy cows (e.g., [5,28,29]). It is a painful condition that impairs the movement of the cow and has detrimental effects on production, reproduction, and longevity [45,75,76]. Another major health disorder in dairy cows is mastitis, due to its high prevalence and impact on milk production [5,75] and longevity [76,77]. Mastitis is an infection of the udder in lactating cows [5]. Due to its high prevalence [5,78], we decided to treat mastitis (in this overview no distinction is made between clinical and subclinical mastitis) as a separate welfare issue (from other infectious diseases). Other welfare issues that received much attention are undernutrition, specifically negative energy balance (NEB), which is often an issue around parturition when feed intake is reduced and milk production rises (e.g., [32,38]), and heat stress, which becomes increasingly more important due to global warming [39]. Both NEB and heat stress are important risk factors for other health issues, such as metabolic disorders (caused by NEB; e.g., [43,55]) and lameness (caused by heat stress; e.g., [39,46]). Similar to mastitis, we decided to treat heat stress as separate issue (from other types of stress), because of the amount of attention it received. Although other health disorders have received less attention in comparison, they may be equally detrimental to cow welfare. For instance, due to few overt symptoms the incidence and severity of metritis (an infection of the uterine wall) may be underestimated [5] and while dystocia (difficulty during calving) may be less prevalent than e.g., lameness [78], it considerably affects the welfare of single cows, since it is considered very painful and increases the likelihood of culling [77,79].

In the reviews, seven welfare issues were identified related to the concern of affective state: pain, stress, negative emotions, discomfort, hunger, thirst, and positive emotions. These welfare issues are not specific to dairy cows, but instead are common to all captive animals, since they are addressed by four of the five freedoms for animals under human control, i.e., freedom from hunger and thirst, freedom from discomfort and freedom from fear and stress and the freedom from pain, injury or disease (the fifth being the freedom to express normal behavior, which addresses the concern of natural living [80]). Although positive emotions were not addressed by the five freedoms, it is now generally accepted that good welfare cannot be simply accomplished by preventing negative affect, but that it also requires the promotion of positive emotions [81]. 

Finally, three welfare issues were identified related to the concern of natural living: the disruption of natural behavior (e.g., lying, movement, feeding), disruption of social environment (e.g., regrouping, large herd size, cow–calf separation, and isolation) and aggression. The first two welfare issues are probably least understood, since, although the concern of natural living is instinctively understood by many, it is difficult to determine which aspects of this natural live are important to the animals [5]. In contrast, aggression is rather a consequence of conflicts between natural behavior and the captive live, such as happens with constant regrouping [29,82]. While each welfare issue would need to be considered to ensure good welfare, it is also important to keep in mind that these welfare issues are extremely interlinked and failure to address one impaired welfare issue can induce a chain of impaired welfare issues. For instance, preventing cows to lie down can lead to stress [5,29], leading to reduced immune functioning [67,70], infectious disease [36], and so on. Ultimately, this also affects reproduction, milk production, and mortality, making these issues not just relevant for cow welfare, but also for economic reasons [83]. 

## 4. Indicators of Cow Welfare

The literature research did not render reviews on dehydration, negative emotions, hunger, thirst, and aggression (see Table S1). However, reviews on other welfare issues sometimes also provided indicators for these issues, allowing to still make some (limited) overview. From the reviews in total 76 different indicators, supported by in total 1143 references, were extracted. These indicators could be categorized as being either environment—or animal—based. In Tables S2 and S3 a full overview is provided of the 76 indicators and their associations with single welfare issues. For space considerations some (closely related) welfare issues were combined, i.e., undernutrition with dehydration and hunger with thirst.

### 4.1. Detection of Reduced Welfare in General

As mentioned in the introduction, the first requirement of an integrated automatic assessment system is to accurately detect as many of incidences of reduced welfare as possible. To determine which indicators may be most helpful to achieve this (generalist indicators), Table 2 shows the indicators that have associations with four or more single welfare issues and that are ranked based on the number of welfare issues they associate with. In addition, it shows the comparison between reports of positive or negative associations across all welfare issues, which gives an indication of whether a clear direction exists across the different welfare issues. In this Table, it is apparent that feed intake shows the broadest and most consistent pattern across the different welfare issues, always indicating a negative association. Thus, reduced feed intake seems to be a reliable generalist indicator of reduced welfare. Although in this overview we did not distinguish between input and output indicators, feed intake was mentioned as a risk factor for some issues (e.g., reduced feed intake around parturition leads to NEB and metabolic disorders [32,38]), while for others it was mentioned as an output (e.g., during heat stress cows reduce their feed intake to reduce the metabolic heat caused by digesting roughage [16,84]). Automatic methods to measure feed intake (e.g., individual electronic scales) are still considered costly and labour intensive and therefore not suited for commercial farms [85]. A good alternative might be to measure feeding time, since this can be measured with less costly systems, e.g., chewing activity monitors [86]. Feeding time has been reported to correlate highly with feed intake [87] and was found here to show a similar consistent pattern across welfare issues. Milk yield also shows an almost consistent pattern across welfare issues, with decreased milk production usually indicating reduced welfare (see Table 2). However, the reports of positive associations reflect that high milk yield is also a risk factor for certain welfare issues, such as NEB (e.g., [88,89]). Despite of its fairly consistent association across welfare issues, many have argued against using milk yield as a welfare indicator, since milk yield is known to vary also due to many other (non-welfare-related) factors and, therefore, may only be used if other indicators already point in the direction of reduced welfare (e.g., [5,90]). 

While physical activity, body condition and lying may also show associations with many different welfare issues, these fail to show a consistent pattern across the welfare issues. This means that an increase or decrease in these indicators cannot be directly interpreted as reduced or improved welfare. In the case of body condition, this seems to be clearly due to opposite associations with different welfare issues, as it has a significant positive association with impaired biological functioning in general and metabolic disorders specifically and a significant negative association with lameness. This is because prepartum over-conditioning can, e.g., depress feed intake and increase liver fat content [35], while thin cows also have thin digital cushions that are less able to absorb shocks during walking, resulting in lameness [44]. Therefore, body condition could play an important role for differentiating between welfare issues (requirement 2). As a generalist indicator body condition may seem less suitable, but it has been suggested that a strong diversion from the ideal body condition score of 3–3.25 (on a five-point scale) could be considered a risk factor of poor welfare [91], meaning that if automatic assessment systems would measure in terms of diversion from this ideal score, body condition could still be a valuable generalist indicator. For lying and physical activity there were often not enough references to determine a clear association with single welfare issues. However there seems to be a distinction between health disorders that trigger a reduction of general activity and negative affective states that trigger more restless behavior. This may be because the studies referenced for negative affect often focused on short-term negative affect, such as negative emotions due to rough handling during milking [92], while diseases are usually accompanied with more long-term negative affective states, such as lethargy and depression, as part of a sickness response that also includes reduced activity [93]. In accordance, our overview shows an inverse association for lying (i.e., increase due to disease and decrease due to negative affect), with the exception for heat stress and mastitis (though not significantly according to the binomial test). During heat stress, cows are suggested to rest more while standing (instead of lying) to increase the body surface available for cooling [94], while cows infected with clinical mastitis may find it painful to lie on the infected udder (e.g., [95,96]).

Other possible suitable generalist indicators, in addition to feed intake (and feeding time) and milk yield, seem to be mainly behavioral indicators (rumination, standing, agonistic behavior, vocalizations, abnormal behavior, and posture). However, there are also several suitable environmental (ambient temperature, humidity, and slippery floors) or physiological (body temperature and heart rate) indicators. For vocalizations it is important to note that the association with welfare is not straightforward [61]. For instance, while many studies reported an increase in vocalizations in negative situations (e.g., [97,98]), there are also specific vocalizations associated with positive situations (e.g., [99]). Therefore, it is more informative to consider the acoustic properties [100,101] and, e.g., distinguish between high vs. low frequency calls as indicators of negative vs. positive states respectively [61]. Also, some caution is warranted for relying solely on body posture as an indicator, since it may only be affected by severe cases of mastitis and lameness [49] and, therefore, would not be useful in an early warning system. The selection of suitable generalist indicators should aim to cover all welfare concerns (biological functioning, affective state and natural lives). This could be achieved either by focusing on indicators associating with all three concerns (e.g., abnormal behavior, feed intake, rumination) or by combining indicators to cover all three concerns. For instance, ambient temperature, which primarily targets biological functioning, could be combined with vocalizations (primarily targeting affective state) and abnormal behavior (primarily targeting natural living).

### 4.2. Differentiation Between Welfare Issues

**Figure 1:**
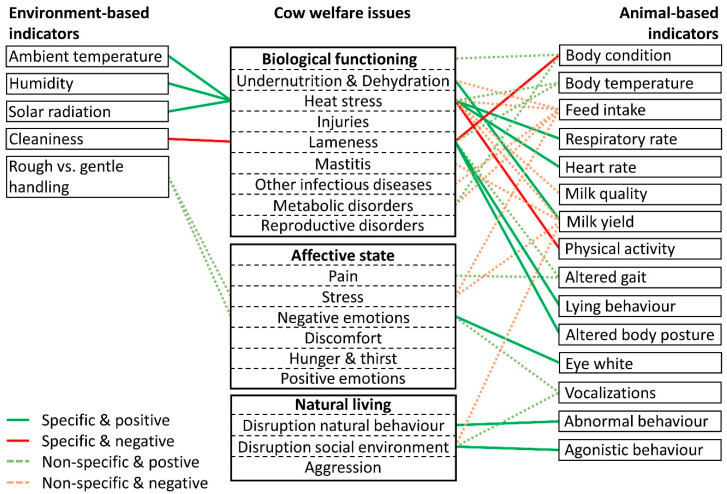
Indicators that have a significant (binomial test, *p* < 0.05) direction of association with single cow welfare issues (middle). Indicators are categorized as either environment-based (left) or animal-based (right). The welfare issues are categorized according to the welfare concern they relate primarily too. If the indicator has no similar association (in terms of direction) with other welfare issues, the association is indicated by a straight line (specific). If a similar association occurs also with another welfare issue, the association is indicated by a dotted line (non-specific). Positive associations are indicated by green lines and negative associations by red lines.

The second requirement of an integrated welfare assessment system is to differentiate between the different welfare issues to give a clear advice to the farmer. To understand which indicators may be suitable for achieving this, Figure 1 provides an overview of all indicators that have a significant positive or negative association with single welfare issues. As mentioned in the introduction, one way to differentiate between welfare issues could be by combining two moderately generalist indicators. However, the overview in Figure 1 shows that this is not easily achieved in the case of dairy cows. Milk yield and feed intake are too general, covering almost all welfare issues, while some less-general indicators—e.g., vocalizations or body temperature—are often not overlapping. One possible combination of generalist indicators would be to use increased body temperature in combination with increased ambient temperature to distinguish heat stress from lameness, which is characterized by increased body temperature in combination with increased lying behavior. Combining generalist indicators could reduce the number of necessary indicators to detect all welfare issues; however, this still only applies to a few welfare issues. Moreover, caution should be taken, since a lack of a significant association may not mean that there is no association. For instance, some sources also suggest a positive association between ambient temperature and lameness [43] and the lack of a significant association may be due to lack of references to support this association. Thus, care should be taken not to make false conclusions based on indicator combinations. 

A second way to differentiate between welfare issues is to select specialist indicators for each single welfare issue, meaning that these indicators have an association with a particular issue that is not shown with other welfare issues. In Figure 1, these specific associations are indicated by solid lines. Immediately, it becomes clear that heat stress and lameness both have several specialist indicators to choose from, while most other welfare issues lack specialist indicators. Since lameness and heat stress were also covered by the most reviews (Table S1), this suggests that for most welfare issues the number of reviews found (possibly reflective of the research done on these topics) was insufficient to identify clear well-established indicators. In addition, it may be more difficult to identify specific indicators to broader welfare issues, such as metabolic disorders. At the moment, splitting these broad issues into more specific issues would not result in a better overview, since not enough references were found for specific metabolic disorders (e.g., ketosis and fatty liver). However, once more information is available on these specific issues, it would be better to treat these issues separately. Figure 1 also shows specialist indicators for undernutrition (milk yield), negative emotions (eye white), disruption of natural behavior (abnormal behavior), and disruption of social environment (agonistic behavior). However, some cautions are necessary for most of these indicators. First, the positive association between milk yield and undernutrition (while all other welfare issues associate negatively with milk yield) is based on reports suggesting that increased milk yield post-partum increases the incidence of NEB, especially in high yielding cows (e.g., [88,89]). However, since this (as well as increased feed intake; e.g., [26,32]) only indicates the risk of undernutrition and is not supported by well-established output indicators, it is still difficult to reliably differentiate undernutrition from other welfare issues. Second, although eye white shows a clear positive association with negative emotions, Ede and colleagues [61] reviewed also evidence that eye white also increases in some positive situations (e.g., [102]) and argued that it may be an indicator of affective arousal, rather than of negative emotions. Finally, abnormal behavior may not be considered an ideal specialist indicator of disruption of natural behavior, since it is also a generalist indicator (Table 1) and shows non-significant associations with other welfare issues in the same direction. However, it should be noted that these other welfare issues, i.e., undernutrition and dehydration, negative emotions, hunger, and thirst, as well as disruption of natural behavior, all could be considered closely linked, since all may be caused by an insufficient feeding regime. Curiously, somatic cell count (SCC) could not be identified as a well-established specialist indicator of mastitis, even though somatic cell count commonly is used to diagnose mastitis (e.g., [103]). The reason for this omission could be that since SCC is already a well-established indicator, the reviews did not consider it noteworthy. Also surprising is that body condition showed no significant association with undernutrition / NEB, which may be due to the reviews mainly focusing on the risk factors of NEB and not so much on any outcome indicators. The absence of well-established indicators for most welfare issues may reflect a lack of research into these issues (although it has to be noted that we used a rather conservative approach for identifying indicators, see Section 6). In the current overview the following indicators are a few supporting studies short of a significant association with a welfare issues and, therefore, may be good candidates for further research: skeletal (or body) size of the cow for reproductive disorders, heart rate for pain or stress, milk flow rate for stress, and ear postures for positive emotions. 

## 5. Opportunities and Challenges for Integrated Automatic Assessment System

The previous section shows that there are several well-established indicators of dairy cow welfare, providing a good (though still not perfect) base for the development of an integrated automatic assessment system. By combining indicators, the system would be able to provide a broader and more detailed welfare assessment than systems based on a single source. Another advantage of an integrated system is that it also could account for more complex associations between indicators and welfare issues. For instance, heat stress is well known to increase when high humidity is combined with high ambient temperature (reviewed by e.g., [15,39]). However, when high humidity (in the form of sprinkler systems) is combined with increased air velocity (in the form of ventilation), it could have a cooling effect [15]. Also, slippery or manure-covered floors affect the gait of cows, which may lead to a false detection of lameness if this factor is not considered [44,104]. A further opportunity to improve the detection accuracy of an integrated automatic assessment system would be to integrate not only sensor data, but also non-sensor information, such as herd data [26,105]. For instance, information on days in milk, parity and previous incidence of a specific welfare issue could all be helpful to identify individuals that would be more at risk to suffer from certain welfare issues (e.g., [17,18]). 

While integrated automatic assessment systems provide many opportunities, there are also a few considerations necessary for measuring the selected indicators. The first consideration is that not all indicators of the same welfare issue would occur simultaneously. Some indicators that can be considered input indicators, such as reduced feed intake for undernutrition, would logically already be detected before a welfare issue arises, while output indicators, such as an altered gait for lameness, could only be detected at the moment or after the welfare issue arises. In this review, we decided not to categorize the indicators as either input or output indicators, since this was usually not clearly mentioned in the reviews. Also, for an automatic assessment system the temporal association is of more interest than the cause-effect relationship. The temporal association can vary both in order (before vs. after) and distance (long or short), depending not only on the causal relationship, but also on how quick the causal effect is. For instance, reduced antepartum feed intake can identify cows that develop metritis a few weeks later (e.g., [106]), while high temperature and humidity induce physiological responses to heat stress within one day [107]. Also, heart rate increases within minutes during stressful events [108], while regrouping may affect milk yield one day to several weeks after it occurred [29]. Unfortunately, also the temporal association was often not mentioned in the reviews, making it more difficult to understand how different indicator measurements should be combined. In any case, an integrated automatic assessment system should be able to store indicator measurements for a considerable time and to be able to retrieve this information if necessary. 

Another important consideration that would need to be made is the setting of reliable thresholds for generating alerts [19]. The literature provides some suggestions for standard thresholds, especially for some indicators related to heat stress. For instance, thresholds for a temperature–humidity index (THI; an index that increases with increasing temperature and humidity) have been suggested at values ranging from 68–74 (reviewed in [16,39,109]). Further suggestions for thresholds have been made for core body temperature (39.4 °C [110]), skin temperature (35 °C [111]), and for respiratory rate (60–90 bpm [111,112]). Also, for SCC as an indicator of mastitis thresholds have been suggested, ranging from 100,000–400,000 cells/mL (e.g., [113,114]). However, for most animal-based indicators, the high inter- and intraindividual variation requires the use of a cow-dependent model with a dynamic threshold that would be based on changes compared to previous measurements on the same individual (e.g., [105,115]). This therefore requires the system to determine the range in which variation in a certain measure is considered ‘normal’ and to generate an alert at a certain deviation from the normal variation. These thresholds are often determined empirically to optimize system performance [116,117,118].

## 6. Discussion

The present review provides a roadmap for the first steps in the development of an integrated automatic assessment system (i.e., identifying welfare issues and suitable indicators), using the example of dairy cows. Although dairy cow welfare has been fairly well-studied over the last decades, there is still a need to know more on many welfare issues. For instance, while some issues have been fairly well-studied (e.g., heat stress), much less is known about how to detect important welfare issues such as dehydration, reproductive disorders, discomfort, positive emotions, thirst, and hunger. While we acknowledge that by focusing on reviews we may have missed some single studies reporting strong associations between an indicator and a welfare issue, we preferred to use a rather conservative approach by focusing on indicators that are supported by many studies and that, therefore, are more likely to show the same association with a welfare issue across different farming regimes, breeds, and measurement methods. Due to a strong positive association between number of studies and number of reviews (e.g., 6, 74, and 394 articles found in Scopus for hunger, pain, and lameness respectively), it may be expected that most of the established indicators have been included in this review. Still, because we used this rather conservative approach by only focusing on reviews and by using the binomial test to select clearly established indicators, we acknowledge that the set of indicators that is selected here could be considered a minimal basis. This means that developers of future integrated assessment systems should not feel limited to use only these indicators, especially if they know an indicator to be reliable for the specific breed of cows or farming regime they are working with.

The review was able to identify many generalist welfare indicators, which means that it may be relatively easy for an integrated automatic assessment system to broaden the detection of reduced welfare by integrating several complementary indicators that could together cover all three welfare concerns. However, it was more difficult to find well-established specialist indicators for most of the welfare issues, making it difficult to differentiate between welfare issues. Also, combining indicators was not found to help much to improve the differentiation between welfare issues. Therefore, future research should focus on clearly establishing more indicators that would allow to differentiate between single welfare issues. Nevertheless, this overview provides enough basis to develop integrated systems that may not yet be able to cover all the different welfare issues, but still would be able to test the added advantage of data integration in praxis. As mentioned in the introduction, some manufacturers already offer systems that integrate several parameters. Such systems could provide a good basis for developing a broader integrated system that should then be tested on the farm. For instance, one commercial system that integrates different milk parameters (Del Pro software, DeLaval International AB, Tumba, Sweden) was found to have a high reliability in detecting clinical mastitis [119]. By adding to such a system other detection techniques—e.g., those that focus on indicators of lameness or heat stress—one could make the first steps in developing a more inclusive cow welfare assessment system. 

The next step in the development of an integrated welfare assessment system would be to select the automatic detection methods. This would depend, for instance, on whether automatic detection methods are already developed and validated, on the costs involved, the ease of use and the invasiveness for the animal. This, therefore, also serves to further select between suitable indicators. While for some indicators, such as ambient temperature, low-cost sensors are already widely in use (e.g., silicon diode-based temperature sensors [120]), other indicators, such as abnormal behavior, are more complex and would require a lot of research to develop a reliable automatic detection method. The final step would be the actual assembly of the system and to validate it in different experimental and farm settings. Apart from providing the basis for the development of an integrated automatic assessment system for dairy cows, the approach used for this review might also be applied for selecting welfare indicators for other farmed animals or possibly even other animal species under human control, such as laboratory animals. In the end, though, the actual success of an integrated automatic assessment system would depend on sufficient research into identifying reliable indicators and the availability of valid automatic detection methods.

## 7. Conclusions

To conclude, research on dairy cow welfare has provided several well-established indicators of reduced welfare that could potentially be measured automatically and integrated into one assessment system. There are several indicators that would allow for a general detection of reduced welfare (generalist indicators), while there are a few indicators that would allow to identify single welfare issues (specialist indicators). Therefore, although the assessment of single welfare issues would need to be improved, this overview allows for the development of prototype integrated automatic assessment systems to assess dairy cow welfare on the farm. In the end, this would help to provide the farmer with a detailed early warning system, allowing him/her to avoid the development of more serious welfare problems.

## Figures and Tables

**Table 1:** References of the reviews that were read for each welfare issue (in brackets). The value after each review refers to the number of new references (i.e., references to articles that were not found in previous reviews; reviews were read in reverse chronological order) they provided as support for associations between indicators and their respective welfare issue.

Welfare Issue	Reviews with No. of New References
Biological functioning	[26]: 2, [30]: 2, [31]: 4, [32] *: 0, [33]: 0, [34] *: 0, [35]: 4, [36]: 6, [37]: 0
Undernutrition	[32] *: 5, [38]: 0
Dehydration	x
Heat stress	[15]: 60, [16]: 66, [39]: 116
Injuries	[40]: 8; [41]: 11
Lameness	[42]: 56, [43]: 48, [44]: 52, [45]: 12, [46] *: 9
Mastitis	[47]: 30, [48]: 23, [49] *: 11
Other infectious diseases	[50]: 7, [51]: 8, [52]: 1, [53]: 0
Metabolic disorders	[54]: 2, [55]: 4, [56]: 0, [57]: 1
Reproductive disorders	[58]: 17, [59]: 62, [60]: 15
Affective state	[61]: 4,
Pain	[62]: 0, [34] *: 1, [49] *: 17, [63]: 0, [64]: 2, [65]: 0, [66]: 0
Stress	[67]: 8, [68]: 24, [69]: 9, [70]: 0
Negative emotions	x
Discomfort	[46] *: 0
Hunger	x
Thirst	x
Positive emotions	[71]: 14
Natural lives	[72]: 0
Disruption natural behavior	[73]: 9
Disruption social environment	[74]: 9

* reviews that covered two welfare issues.

**Table 2:** Indicators that are associated with four or more welfare issues, ranked according to the number of welfare issues they were reported to have associations with. No. references refers to the total number of references reporting an association for each indicator. ↑/↓ indicates the total number of references indicating a positive association versus the total number of references indicating a negative association. Significant differences between the number of positive and negative associations are highlighted in bold (binomial test, *p* < 0.05). No. significant positive/negative associations refers to the number of welfare issues that show either a significant (binomial test, *p* < 0.05) positive or negative association with each indicator. This number can be derived from Tables S2 and 3 where significant associations between welfare issues and indicators are highlighted in bold.

Environment/Animal Based	Indicator	No. Welfare Issues	No. References	↑/↓	No. Significant Positive Associations	No. Significant Negative Associations
animal	feed intake	16	102	**9/91**	0	5
animal	milk yield	15	155	**36/102**	1	4
animal	lying behavior	12	50	26/21	1	0
animal	physical activity	12	38	17/19	0	1
animal	rumination	11	27	**2/24**	0	0
animal	body condition	10	87	35/24	2	1
animal	body temperature	10	61	**53/0**	2	0
animal	feeding time	10	23	**1/22**	0	0
animal	heart rate	9	25	**24/0**	1	0
animal	standing behavior	9	25	**20/1**	0	0
animal	agonistic behavior	7	20	**16/4**	1	0
animal	vocalizations	6	33	**30/0**	2	0
animal	milk quality/content	6	26	**2/12**	0	1
animal	body weight	6	10	**0/9**	0	0
environment	ambient temperature	5	40	**30/3**	1	0
animal	somatic cell count in milk	5	12	**10/0**	0	0
animal	feeding speed	5	8	4/4	0	0
animal	social behavior (unspecified)	5	6	2/4	0	0
environment	humidity	4	28	**23/1**	1	0
animal	abnormal behavior	4	21	**21/0**	1	0
animal	altered body posture	4	21	**18/0**	1	0
environment	wet/slippery floors	4	19	**10/1**	0	0
animal	feeding frequency	4	10	2/8	0	0
animal	locomotion behavior	4	10	2/7	0	0
animal	milking frequency	4	9	6/3	0	0
animal	grooming	4	9	4/4	0	0
animal	elimination	4	6	5/0	0	0

## Supplementary Materials

The following are available online at https://www.mdpi.com/2076-2615/10/8/1430/s1, Table S1: list of search terms and resulting number of review finds, Table S2: The number of references supporting each association between indicators and welfare issues related to biological function, as well as the number of references identifying either a positive or negative association (this excludes references not mentioning a direction of association or that suggest a less straight forward association), Table S3: The number of references supporting each association between indicators and welfare issues related to affective state and natural living, as well as the number of references identifying either a positive or negative association (this excludes references not mentioning a direction of association or that suggest a less straightforward association).
